# Autism-associated R451C mutation in neuroligin3 leads to activation of the unfolded protein response in a PC12 Tet-On inducible system

**DOI:** 10.1042/BJ20150274

**Published:** 2016-02-09

**Authors:** Lisa Ulbrich, Flores Lietta Favaloro, Laura Trobiani, Valentina Marchetti, Vruti Patel, Tiziana Pascucci, Davide Comoletti, Stefan J. Marciniak, Antonella De Jaco

**Affiliations:** * Department of Biology and Biotechnology “Charles Darwin” and Pasteur Institute - Cenci Bolognetti Foundation, Sapienza University of Rome, Piazzale Aldo Moro 5, 00185 Rome, Italy; † Department of Internal Medicine, University of Rome Tor Vergata, Via Montpellier, 100133 Rome, Italy; ‡ Department of Psychology, “Daniel Bovet” Neurobiology Research Center Sapienza University of Rome, Santa Lucia Foundation, European Center for Brain Research CERC, via dei Marsi 78, 00185 Rome, Italy; § Department of Neuroscience and Cell Biology and Department of Pediatrics, Child Health Institute of New Jersey, Rutgers, Robert Wood Johnson Medical School, 125 Paterson Street, New Brunswick, NJ 08901, U.S.A.; ║ Department of Medicine, Cambridge Institute for Medical Research, University of Cambridge, Hills Road, Cambridge CB2 0XY, UK.

**Keywords:** autism, ER stress, molecular chaperones, neuroligin, protein misfolding, unfolded protein response

## Abstract

Several forms of monogenic heritable autism spectrum disorders are associated with mutations in the neuroligin genes. The autism-linked substitution R451C in neuroligin3 induces local misfolding of its extracellular domain, causing partial retention in the ER (endoplasmic reticulum) of expressing cells. We have generated a PC12 Tet-On cell model system with inducible expression of wild-type or R451C neuroligin3 to investigate whether there is activation of the UPR (unfolded protein response) as a result of misfolded protein retention. As a positive control for protein misfolding, we also expressed the mutant G221R neuroligin3, which is known to be completely retained within the ER. Our data show that overexpression of either R451C or G221R mutant proteins leads to the activation of all three signalling branches of the UPR downstream of the stress sensors ATF6 (activating transcription factor 6), IRE1 (inositol-requiring enzyme 1) and PERK [PKR (dsRNA-dependent protein kinase)-like endoplasmic reticulum kinase]. Each branch displayed different activation profiles that partially correlated with the degree of misfolding caused by each mutation. We also show that up-regulation of BiP (immunoglobulin heavy-chain-binding protein) and CHOP [C/EBP (CCAAT/enhancer-binding protein)-homologous protein] was induced by both mutant proteins but not by wild-type neuroligin3, both in proliferative cells and cells differentiated to a neuron-like phenotype. Collectively, our data show that mutant R451C neuroligin3 activates the UPR in a novel cell model system, suggesting that this cellular response may have a role in monogenic forms of autism characterized by misfolding mutations.

## Abstract (precis)

The expression of the autism-related mutant R451C neuroligin3 activates the three branches of the unfolded protein response in neuronal-like PC12 cells and leads to up-regulation of the response target genes BiP and CHOP.

## INTRODUCTION

Genetic studies of monogenic forms of ASDs (autism spectrum disorders) have identified synaptic function as one of the molecular pathways underlying neurodevelopmental disorders [1]. Among the most studied susceptibility genes are the neuroligins (NLGNs), postsynaptic cell adhesion proteins involved in the maturation, specification and plasticity of neuronal networks through the interaction with presynaptic neurexins [2]. Rare human autism-linked mutations in the NLGN genes have been shown to affect protein folding and trafficking to the cell surface.

The R451C single point mutation in *NLGN3*, an X-linked gene, was found in association with highly penetrant autism in a Swedish family [3]. The R451C NLGN3 protein has been extensively characterized in overexpression studies in several systems [4–7]. Partial retention in the ER (endoplasmic reticulum) of the R451C mutant protein resulted from local misfolding of the extracellular domain of NLGN3. Whereas most of the mutant protein was degraded, a small fraction of the protein was correctly trafficked to the cell surface [8]. Consistent with these *in vitro* studies, *in vivo* work on knockin R451C NLGN3 mice has shown that this mutation caused a 90% reduction in NLGN3 protein levels [9].

A group of diseases called ERSDs (ER storage diseases) includes disorders characterized by protein misfolding and its recognition by the ER quality control system [10]. For these disorders, the pathological phenotype may be due to ER retention of excess misfolded protein and/or to the lack of functional protein at the final destination. ERSDs are characterized by the presence of ER stress due to protein overload, and by the activation of an adaptive and protective response called the UPR (unfolded protein response), finalized to restore normal ER function [11,12]. ER stress triggers the UPR through three sensors present in the ER membrane: IRE1 (inositol-requiring enzyme 1), PERK [PKR (dsRNA-dependent protein kinase)-like endoplasmic reticulum kinase] and ATF6 (activating transcription factor 6), which are normally maintained in the inactive conformation by association to the molecular chaperone BiP (immunoglobulin heavy-chain-binding protein) [13]. Downstream targets of the UPR include genes encoding chaperones, molecules involved in ERAD (ER-associated degradation), membrane remodelling and protein trafficking [14].

**Figure 1:**
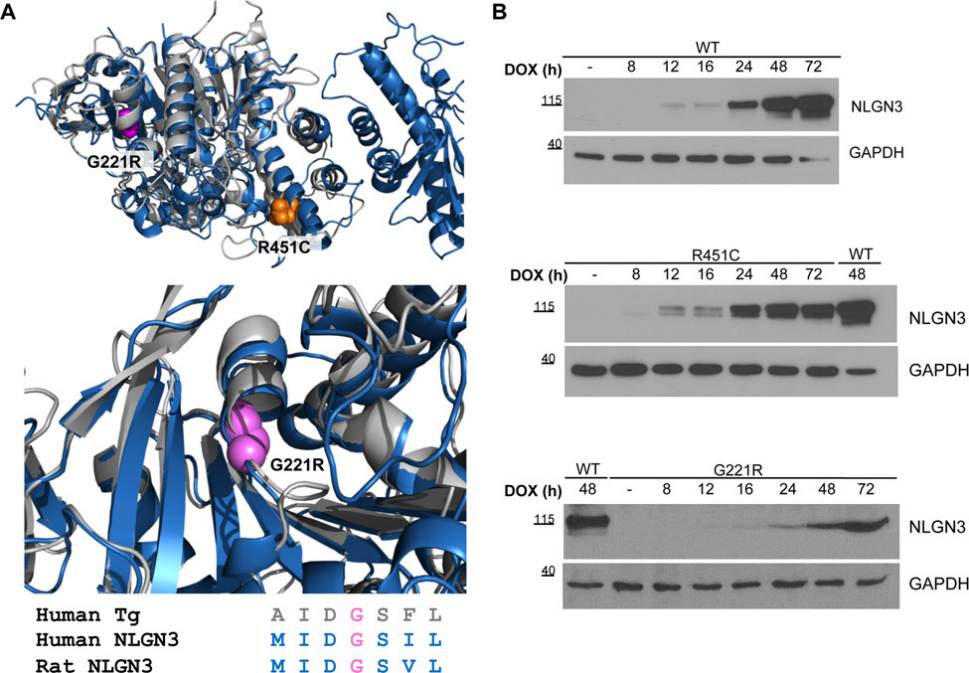
Structure and time course of expression of NLGN3 in inducible PC12 Tet-On clones (**A**) Upper panel: overlay of the three dimensional structure of the α/β-hydrolase domain of NLGN4 (blue, PDB code 3BE8) and the homology model of the cholinesterase-like domain of Tg (grey), with the location of the R451C (orange) and G221R (magenta) mutations. Overlay was obtained using the program PyMOL (http://www.pymol.org). The Swiss Model Server (http://swissmodel.expasy.org/) was used to obtain structural template for the cholinesterase-like domain of Tg (residue 2192 to residue 2719). Lower panel: magnification of the homologous region between NLGN4 and Tg where G221R/G2300R are located. Sequence alignment shows that the glycine residue is highly conserved in NLGN3 and Tg. (**B**) NLGN3 expression in PC12 cells after DOX treatment (0–72 h). WT lysates at 48 h from DOX were used as a reference for migration and band intensity across the three panels. Molecular masses are indicated in kDa.

The goal of the present study was to understand whether the autism-associated R451C NLGN3 protein elicits ER stress and UPR activation in mammalian cells. We undertook a detailed characterization of the three UPR signalling branches in a new model system consisting in PC12 Tet-On cells with stable and inducible expression of WT (wild-type), R451C or G221R NLGN3. The G221R substitution in NLGN3 has been used as a positive control because it was shown previously to severely disrupt the folding of the extracellular domain of NLGN3 and to cause a virtually complete retention of the protein within the ER [8]. Although this mutation has not been described in NLGN3, the G2300R substitution in thyroglobulin (Tg) is associated with hypothyroidism, and causes defective intracellular protein transport and retention within the ER [15]. The affected Gly^2300^ of Tg is homologous with Gly^221^ in NLGN3, and is conserved in both proteins across different species, mapping to the core of the α/β-hydrolase domain of the NLGNs that is structurally similar to the C-terminal portion of the Tg protein [16] (Figure 1A).

Our data provide strong evidence that retention of NLGN3 caused by the R451C and G221R mutations induces the activation of the UPR, albeit with different intensities and timing profiles that partially correlate with the degree of misfolding caused by each mutation on NLGN3. We show that all three ER stress sensors, ATF6, IRE1 and PERK, are activated by the mutant R451C NLGN3 protein, eliciting the corresponding signalling cascades in a time-dependent manner upon NLGN3 synthesis. Up-regulation of BiP and CHOP [C/EBP (CCAAT/enhancer-binding protein)-homologous protein] was detected in both undifferentiated and differentiated PC12 cells, supporting the hypothesis that ER stress and UPR signalling induced by misfolded proteins might influence neuronal functioning in individuals carrying the mutation.

## MATERIALS AND METHODS

### Reagents and antibodies

Reagents, buffers, culture media and serum for cell cultures were from Sigma–Aldrich unless stated otherwise.

The following commercial antibodies were used: anti-NLGN pan-mouse monoclonal antibody (clone 4F9, catalogue number 129-011, Synaptic Systems), anti-p-eIF2α (eukaryotic initiation factor 2α) (Ser^51^) rabbit antibody (119A11, Cell Signaling Technology), anti-eIF2α total mouse antibody (L57A5, Cell Signaling Technology), anti-BiP rabbit polyclonal antibody (ab53068, Abcam), anti-CHOP mouse monoclonal antibody (ab11419, Abcam), rabbit polyclonal anti-GAPDH (glyceraldehyde-3-phosphate dehydrogenase) (ab37168, Abcam), goat polyclonal anti-lamin A/C (N18, Santa Cruz Biotechnology), mouse monoclonal anti-KDEL (ADI-SPA-827, Enzo Life Sciences), mouse βIII-tubulin (MMS-435P, Covance), rabbit anti-FLAG (F7425, Sigma), mouse anti-FLAG M2 monoclonal antibody (F3165, Sigma) and rabbit anti-calnexin polyclonal antibody (SPA-860, Enzo Life Sciences). Goat secondary anti-rabbit IgG (rhodamine-conjugated) and anti-mouse IgG (FITC-conjugated) were from Abcam.

### Plasmid construction

pTRE tight Tet-On containing the N-terminal FLAG-tagged full-length rat *NLGN3* cDNA was created by inserting the *NLGN3* cDNA extracted from pcDNA3.1-NLGN3 [4] in the Tet-On pTRE tight vector (Clontech). The luciferase reporter constructs used were the following: (5×)ATF6-luc (firefly) encoding five tandem copies of the ATF6 consensus binding sites upstream of the luciferase gene; and the pRL-TK (*Renilla*), which drives *Renilla* luciferase constitutive expression as a reporter for transfection efficiency. Both luciferase constructs were originally from Dr Timothy Weaver (Cincinnati Children's Hospital Medical Center, Cincinnati, OH, U.S.A.) [17].

### Culture of stable PC12 Tet-On cells

PC12 Tet-On cells were cultured following the supplier's guidelines (Clontech). Briefly, cells were cultured in DMEM (Dulbecco's modified Eagle's medium) supplemented with 10% (v/v) horse serum, 5% (v/v) Tet-approved FBS (BD Biosciences), 10 mM Hepes (PAA), 1× GlutaMAX™ (Gibco), 0.2 unit/ml bovine insulin, 200 μg/ml Geneticin®, 150 μg/ml hygromycin B (Life Technologies).

PC12 Tet-On cells were differentiated into neurons by plating on to glass coverslips pre-treated with 0.1 mg/ml poly-L-lysine and 0.1 mg/ml rat tail collagen I, and culturing in DMEM supplemented with 1% (v/v) horse serum, non-essential amino acids, Hepes, 0.2 unit/ml bovine insulin, 200 μg/ml Geneticin®, 150 μg/ml hygromycin B and 150 ng/ml NGF (nerve growth factor) for 5 days. Tunicamycin treatments of PC12 Tet-On parental cells, used as positive control for UPR activation, was performed for 16 h at 2 μg/ml. Thapsigargin was used at 500 nM for 16 h.

### Generation and characterization of stable PC12 Tet-On cell lines expressing NLGN3

PC12 NLGN3 Tet-On clones were generated by co-transfecting pTRE response plasmid encoding NLGN3 and pTK-Hyg resistance vector, using the Lipofectamine™ procedure (Life Technologies). Transfected cells were cultured in a selective medium containing 150 μg/ml hygromycin B and 200 μg/ml Geneticin®. Isolated clones were screened for NLGN3 expression by Western blot analysis after NLGN3 expression was induced with doxycycline (DOX). NLGN3 expression was typically induced with 1 μg/ml DOX. The MG132 proteasome inhibitor was added in the medium for the last 16 or 24 h of culture, depending on the experimental procedures at concentrations ranging from 0.1 to 1 μM. GSK2606414 (Selleck Chemicals) treatment (50 nM for 60 min) was used to evaluate PERK-mediated phosphorylation of eIF2α.

### Preparation of PC12 Tet-On cell extracts

Lysates of PC12 cells were prepared using lysis buffer (150 mM NaCl, 10 mM Tris/HCl, pH 8.0, and 0.5% Nonidet P40) supplemented with protease inhibitor cocktail. Detection of eIF2α phosphorylation required a lysis buffer with phosphatase inhibitors (10 mM tetrasodium pyrophosphate, 15.5 mM 2-glycerophosphate and 100 mM NaF). Lysis was performed for 15 min on ice, and the soluble fraction was cleared by centrifugation at 17000 ***g*** for 15 min at 4°C.

### Nuclear fraction enrichment

The cytosolic fraction was isolated by incubating the cells on ice for 10 min with low-salt lysis buffer (10 mM Hepes, pH 7.4, 42 mM KCl, 5 mM MgCl_2_, 1 mM DTT, 0.5% Nonidet P40 and protease inhibitor cocktail) followed by centrifugation at 17000 ***g*** for 10 min at 4°C. The supernatant was collected, whereas for nuclear fraction enrichment, the insoluble pellet was further incubated on ice for 30 min with high-salt buffer (50 mM Tris/HCl, pH 7.5, 400 mM NaCl, 1 mM EDTA, 1% Triton X-100, 0.5% Nonidet P40, 10% glycerol, 2 mM DTT and protease inhibitor cocktail), followed by centrifugation at 17000 ***g*** for 10 min at 4°C. The supernatant fraction was used to detect CHOP and lamin A/C in the nuclei.

### SDS/PAGE and Western blotting

Standard techniques for protein analysis included protein quantification using the Bradford assay, protein separation by SDS/PAGE (10% gels) (Bio-Rad Laboratories) in running buffer (25 mM Tris/HCl, 20 mM glycine and 3.5 mM SDS) and transfer on to Immobilon P membranes (Millipore) with transfer buffer [25 mM Tris/HCl, 20 mM glycine and 10% (v/v) methanol]. Membranes were blocked with 5% (w/v) non-fat dried skimmed milk powder in T-TBS (20 mM Tris/HCl, pH 7.6, 137 mM NaCl and 0.2% Tween 20). Blocking solution for detecting eIF2α phosphorylation was 3% (w/v) BSA in PBS. All primary antibodies were used at a 1:1000 dilution in PBS containing 3% (w/v) BSA. Secondary antibodies were diluted in 5% (w/v) non-fat dried skimmed milk powder in T-TBS: HRP (horseradish peroxidase)-conjugated anti-mouse and anti-rabbit were diluted 1:10000 and HRP-conjugated anti-goat was diluted 1:50000. The HRP signal was developed using the LiteAblot PLUS and TURBO extra sensitive chemoluminescent substrates (Euroclone) and exposed to autoradiographic films (Santa Cruz Biotechnology) or revealed by using the ChemiDoc™ MP System (Bio-Rad Laboratories). Densitometry was performed on at least three independent experiments using ImageJ software (NIH).

### Endoglycosidase H digestion

Cell extracts from PC12 NLGN3 Tet-On cells induced for 24 h by DOX were denatured for 10 min using a commercial buffer (New England Biolabs). EndoH (endoglycosidase H) (New England Biolabs) digestion employed 1500 enzymatic units in the provided reaction buffer at 37°C for 3 h. Digested proteins were subjected to SDS/PAGE and immunoblotting using the commercial anti-NLGN antibody.

### Immunocytochemistry

PC12 Tet-On cells expressing either WT or mutant NLGN3 proteins were plated on glass coverslips coated with 0.1 μg/ml poly-L-lysine and grown in DMEM. Cells were fixed in 4% (w/v) paraformaldehyde in PBS, washed and incubated with blocking buffer (PBS, 2% normal goat serum, 0.5% BSA and 50 mM glycine) [4]. Primary antibody incubation was performed with either rabbit anti-FLAG or anti-FLAG M2 mouse monoclonal antibody diluted 1:500 in combination with the anti-calnexin or anti-CHOP diluted 1:200 in 5× diluted blocking buffer. Secondary antibodies were diluted 1:500 in the same buffer. Nuclei were stained with DAPI.

Four or five images for each sample were captured at room temperature using a Nikon inverted confocal microscope with ×40 and ×63 objective lenses and processed using ImageJ and Adobe Photoshop software.

### Real-time RT (reverse transcription)–PCR

Total RNA was extracted from PC12 NLGN3 Tet-On cells by using TRIzol® reagent (Life Technologies) following the manufacturer's procedures. First-strand cDNA was synthesized using Superscript II (Life Technologies) starting from 4 μg of RNA following the manufacturer's recommended protocols. cDNA was amplified by using SYBR Green (Life Technologies) and the Applied Biosystems suggested protocol consisting of 40 cycles of amplification with each cycle including 15 s at 95°C followed by 60 s at 60°C. Real-time RT–PCR was carried out using a thermocycler iCycler PCR Detection System (Bio-Rad Laboratories) and individual melting curves were produced to assess the specificity of each primer set. All assays were run in triplicate and relative quantification was calculated using the comparative *C*_T_ method [18].

Used primer sets were: BiP forward 5′-TACTCGAATT-CCAAAGATTCAG and reverse 5′-TCAAGCAGAACCAG-GTC; XBP1s forward 5′-ATGGATGCCCTGGTTGCTGAA and reverse 5′-CCTGCACCTGCTGCGGACT; 18s forward 5′-ACGGACCAGAGCGAAAGCAT-3′ and reverse 5′-TGT-CAATCCTGTCCGTGTCC-3′; GAPDH forward 5′-GTGCCA-GCCTCGTCTCATAG-3′ and reverse 5′-TGATGGCAACAA-TGTCCACT-3′.

### Luciferase assay

PC12 NLGN3 Tet-On clones were seeded on 0.1 mg/ml poly-L-lysine pre-coated six-well plates. At 24 h after plating, NLGN3 expression was induced for several lengths of time (0, 12, 16, 24, 48 and 72 h) and transfection with Lipofectamine™ 2000 occurred for the last 24 h using 2 μg of total DNA: pATF6(5×)-Luc (firefly) and PRL-TK (*Renilla*) in a 50:1 ratio. Lysis buffer provided by the Dual-Luciferase Reporter Assay (Promega) was used for harvesting the cells and the substrates included in the kit were used for determining firefly and *Renilla* luciferase activities. A GloMax multi+ detection system (Promega), with injectors for substrate addition, was used for detecting the luminescence signals. The luciferase activity was calculated as a ratio of the firefly to *Renilla* value relative to the −DOX condition. All measurements were carried out in triplicate.

### Statistical analysis

All the experiments were performed at least three times on independent samples.

For all experiments, one-way ANOVA followed by Bonferroni's post-hoc test were used for statistical analysis with Prism5 (GraphPad Software): *P<0.05, **P<0.01, ***P<0.001; ns, non-significant.

## RESULTS

### R451C and G221R NLGN3 are retained within the ER and degraded by ERAD in a novel PC12 cell model system

Time-dependent activation of the UPR was studied in the rat phaeochromocytoma PC12 cell line, modified with the Tet-On gene regulatory system. This line was chosen because it can be differentiated into a neuronal-like phenotype by NGF treatment [19]. PC12 Tet-On NLGN3 cell lines were generated by transfecting PC12 cells with the DOX-inducible pTRE-tight vector containing the FLAG-tagged cDNAs encoding WT, R451C or G221R NLGN3.

Stably transfected PC12 clones expressing NLGN3 were screened for DOX-induced NLGN3 synthesis. Clones grown in the absence of DOX were used to assess the stringency of the Tet-On expression system. In addition to the WT protein and the R451C mutant variant that causes autism, we have also created a PC12 G221R NLGN3 line as a positive control for protein misfolding. This mutation introduces a bulky arginine side chain in the core of the α/β-hydrolase domain (Figure 1A), leading to complete misfolding of the extracellular domain of NLGN3, as indicated by previous studies based on the use of trypsin proteolysis [8].

**Figure 2:**
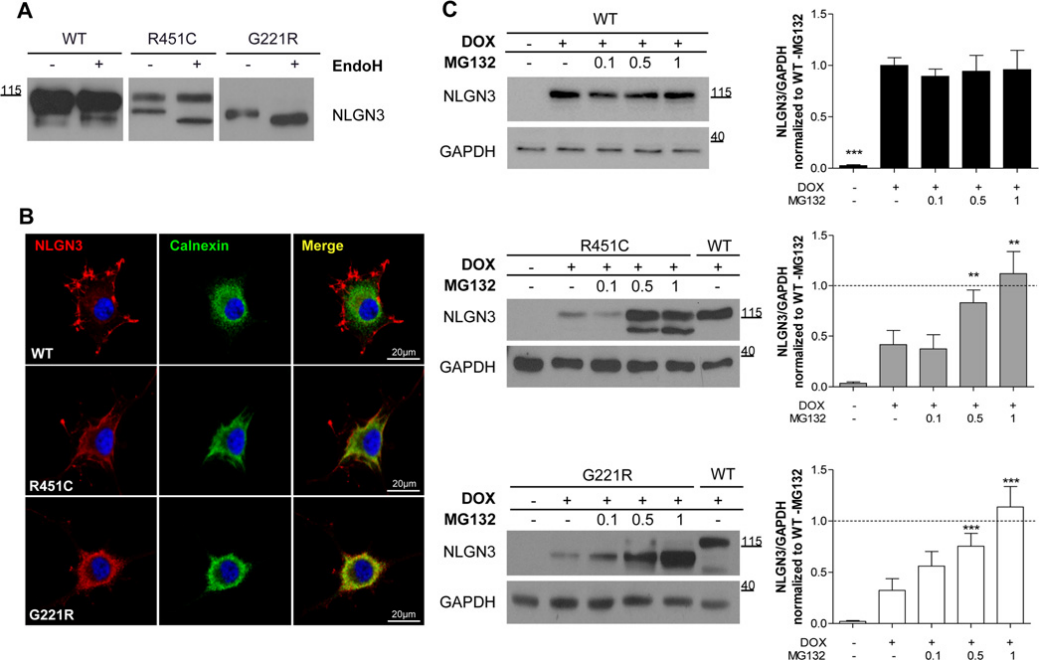
Localization and degradation of NLGN3 in inducible PC12 Tet-On clones (**A**) EndoH treatment of lysates from cells expressing WT, R451C and G221R NLGN3. The image presents different parts of the same Western blot. (**B**) NLGN3 localization in PC12 cells differentiated for 5 days with NGF. WT NLGN3 (FLAG, red) did not show any co-localization with calnexin (green), in contrast with the R451C and G221R mutant proteins showing partial and complete co-localization (yellow). DAPI (blue) was used to stain the nuclei. (**C**) Left panels: Western blot analysis of NLGN3 protein levels after 48 h of DOX treatment and 24 h of MG132 (0.1, 0.5 and 1μM). GAPDH was used as loading control. Histograms on the right show NLGN3/GAPDH densitometric analysis normalized to WT NLGN3 levels in the absence of MG132. Statistical analysis comparing treated and untreated conditions within each clone (*n*=4; **P<0.01, ***P<0.001). Molecular masses are indicated on the blots in kDa.

Three representative clones were chosen for each NLGN3 variant (WT, R451C-2 and G221R-1, Supplementary Figures S1A and S1B), based on NLGN3 protein and mRNA levels. We named the new cell lines WT, R451C and G221R NLGN3. Western blot analysis of the cell lysates was used to investigate the time-dependent expression of NLGN3 in these lines (Figure 1B and Supplementary Figure S1C). In cells expressing WT NLGN3, we detected the presence of a single ∼110 kDa band. In contrast, R451C NLGN3 protein presented as two bands with apparent molecular masses ranging from ∼100 kDa to ∼110 kDa. G221R NLGN3 appeared as a single band of ∼100 kDa (Figure 1B and Supplementary Figure S1C). We then used sensitivity to EndoH to detect the presence of immature high-mannose N-glycans typical of proteins retained in the ER and to characterize the bands observed by Western blotting (Figure 2A). As expected, WT NLGN3 was resistant to enzymatic digestion, as indicated by the absence of a band shift upon EndoH treatment. In contrast, the faster migrating band of R451C NLGN3 showed a band shift after digestion, revealing its immature nature. G221R NLGN3 mutant protein showed a complete digestion to a lower deglycosylated band (Figure 2A) in agreement with the complete retention of this mutant protein in the ER [8]. To confirm that the mutant proteins were retained in the ER, we used immunofluorescence co-localization analysis with the ER marker calnexin. WT NLGN3 localized to the cell membrane, whereas R451C NLGN3 showed partial ER retention and G221R showed complete co-localization with calnexin, supporting our previous observations [7] (Figure 2B).

To ascertain whether the involvement of ERAD might explain the lower levels of protein seen at steady state for the R451C and G221R mutants in comparison with WT NLGN3, we treated the cells with different concentrations of the proteasome inhibitor MG132 (Figure 2C). A significant increase in the levels of the mutant variants of NLGN3 was observed after MG132 treatment (0.5 μM and 1 μM), whereas WT NLGN3 levels remained unaltered. Consistent with our previous findings [8], this indicates that the mutant proteins were degraded by ERAD.

### Mutant R451C and G221R NLGN3 activate the transcriptional arm of the UPR

**Figure 3:**
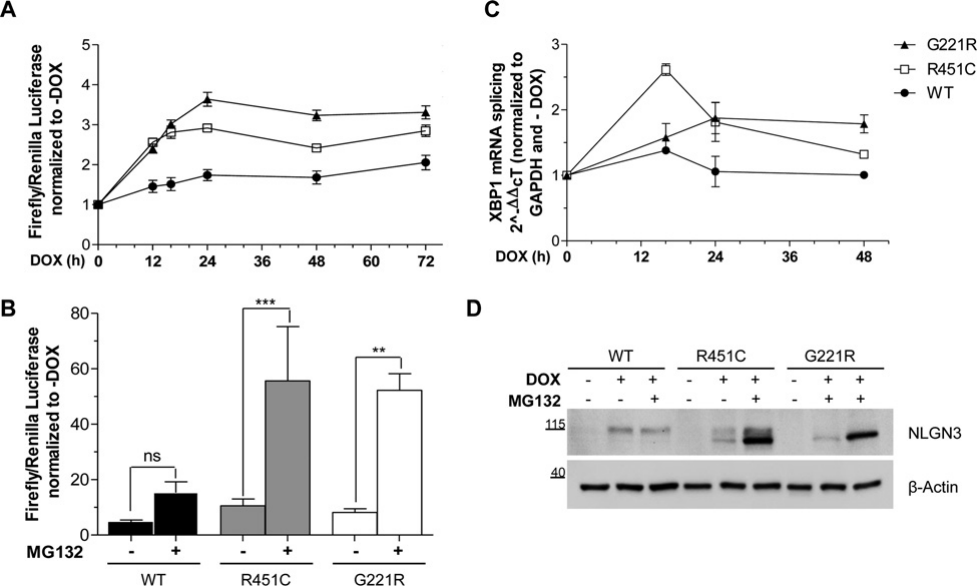
Activation of the UPR transcriptional factors (**A**) PC12 cells were transfected with the luciferase reporter vectors after inducing NLGN3 synthesis with DOX (from 0 to 72 h). For each clone, the ratio of firefly/*Renilla* luciferase was normalized to the −DOX condition at each of the time points. Results are means±S.E.M. for six independent experiments. Mutants compared with WT values, for all time points: ***P<0.001, except for R451C at 48 h, *P<0.05 and 72 h, **P<0.01. R451C compared with G221R at 24 h and 48 h: **P<0.01. (**B**) NLGN3 synthesis was induced for 48 h with DOX and 0.5 μM MG132 was added for the last 24 h. Results are means±S.E.M. for five independent experiments (***P<0.001 and **P<0.01). (**C**) NLGN3 synthesis was induced, from 0 to 48 h, and the splicing of XBP1 was quantified by real-time RT–PCR. Changes in XBP1s were calculated relative to the 18S housekeeping gene and normalized to the −DOX condition. For each time point, statistical relevance of four independent experiments was calculated comparing NLGN3 mutants with WT. A peak value of XBP1s was observed at 16 h from DOX induction in the R451C NLGN3 cells (R451C compared with WT, ***P<0.001; R451C compared with G221R, **P<0.01), whereas G221R NLGN3 overexpression caused a slower but steady XBP1 splicing increase (*P<0.05 at 24 h and **P<0.01 at 48 h). XBP1 splicing in WT NLGN3 cells was unchanged over time. (**D**) NLGN3 protein levels were quantified after DOX induction of NLGN3 synthesis for 16 h in the presence or absence of MG132 (0.5 μM). The Western blot is representative of four independent experiments. Molecular masses are indicated in kDa.

The activation of each UPR branch driven by ATF6, IRE1 and PERK was studied in our cell model, by performing time course experiments. We first analysed the activation of the UPR by using a reporter where luciferase expression is driven by ERSEs (ER stress elements) based on those found in the promoters of ATF6 target genes [pATF6(5×)-Luc] [17]. We co-transfected the PC12 NLGN3 cells with the pATF6(5×)-Luc reporter and the transfection control pKT-*Renilla* at different times after DOX administration (0, 12, 16, 24, 48 and 72 h). Both mutant variants of NLGN3 induced the highest luciferase signal between 12 and 24 h, significantly above the values obtained for the WT NLGN3 cells (Figure 3A). For the mutant proteins, the activation of the reporter remained stable during prolonged NLGN3 synthesis and was significantly different from the WT. The highest levels of ATF6 activation were achieved by the G221R mutant from 24 to 72 h. Because the R451C and G221R mutations increase NLGN3 degradation through ERAD (Figure 2C), we also analysed the activation of this reporter after blocking the proteasome with MG132. A significant increase in signalling was observed only in R451C and G221R NLGN3-expressing cells, but not in WT NLGN3 cells (Figure 3B). These results indicate that R451C and G221R NLGN3 behave as *bona fide* misfolded proteins and that their retention in the ER activates the UPR.

The DNA-binding sites for ATF6 and XBP1 (X-box-binding protein 1) share some similarity and so it is possible that the ATF6(5×)–Luc reporter may reflect IRE1 signalling in addition to ATF6 activation [20]. During ER stress, ATF6 traffics to the Golgi apparatus where it is cleaved to a shorter cytoplasmic fragment that translocates to the nucleus and acts as a transcriptional factor. We thus attempted to study ATF6 activation directly by immunodetection of cleaved ATF6. However, in our hands, none of three different anti-ATF6 antibodies was successful for examining ATF6 processing.

We next investigated the specific activation of IRE1. This ER stress sensor initiates the unconventional splicing of *XBP1* mRNA generating the spliced XBP1 form (XBP1s), which is directly involved in the transcriptional activation of UPR target genes, such as ERAD components [21]. We quantified the levels of *XBP1s* mRNA to evaluate IRE1 activation in our PC12 cell lines after inducing NLGN3 synthesis for specific times (0, 16, 24 and 48 h) (Figure 3C). Real-time RT–PCR revealed time-dependent changes in the levels of XBP1s for the R451C and G221R NLGN3-expressing cells, relative to the condition where NLGN3 synthesis was not induced (−DOX), whereas WT NLGN3 synthesis did not cause significant changes in XBP1s levels at any of the times investigated (Figure 3C). The trend of XBP1 splicing appeared different between the two mutants: the R451C mutant caused an early and strong increase at 16 h from synthesis, followed by its attenuation up to 48 h. The G221R mutant induced a slower but sustained increase in XBP1s levels over time, with the highest levels at 24 and 48 h. Our time course data indicate that NLGN3 protein levels were lower at 16 h than at longer times of induction (Figure 1B). We studied the degradation of mutant NLGN3 at 16 h after DOX induction using the proteasome inhibitor MG132. We observed a striking increase in R451C and G221R protein levels after proteasomal inhibition (Figure 3D), indicating that ERAD prevented the accumulation of these mutant proteins.

### Mutant R451C and G221R NLGN3 activate PERK-mediated eIF2α phosphorylation

**Figure 4:**
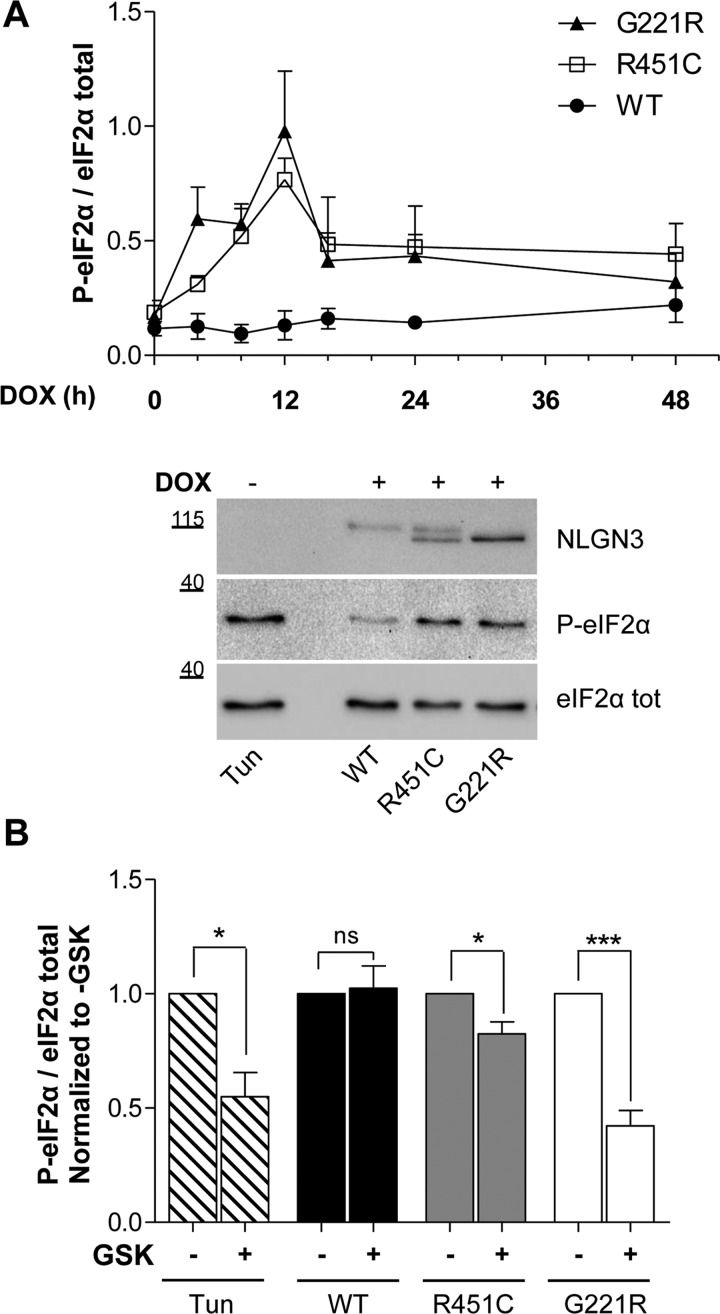
PERK branch activation (**A**) Upper panel: time course densitometric analysis of Western blot for p-eIF2α, from 0 to 48 h from DOX treatment, normalized to total eIF2α levels. Significant differences were observed for R451C and G221R NLGN3 mutant clones at 12 h from DOX treatment (**P<0.01 and ***P<0.001 respectively) in comparison with WT. Mean±S.E.M. values were from four independent experiments. Lower panel: representative Western blot for p-eIF2α and total eIF2α from PC12 NLGN3 Tet-On cells at 12 h after DOX administration. Molecular masses are indicated in kDa. (**B**) Densitometry of p-eIF2α normalized to total eIF2α from four independent experiments on PC12 cells at 12 h from DOX induction, without or with 50 nM GSK2606414 treatment. Mean±S.E.M. values were from four independent experiments (*P<0.05, **P<0.01, ***P<0.001).

The phosphorylation of eIF2α integrates multiple cellular stress pathways [22]. Regulated phosphorylation of eIF2α by the ER stress-activated protein kinase PERK modulates protein synthesis and couples the production of ER client proteins with the organelle's capacity to fold and process them [23]. Since phosphorylation of eIF2α has been shown to occur soon after ER-stress induction in PC12 cells [24], we studied PERK activation upon NLGN3 synthesis after 0, 4, 8, 12, 16, 24 and 48 h. We observed that p-eIF2α levels increased over time with a similar trend for the R451C and G221R mutant variants, whereas they remained unaltered for the WT protein (Figure 4A, upper panel). The increase in p-eIF2α was detectable after 4 h from NLGN3 induction and reached the highest peak at 12 h, with both mutant variants reaching significantly higher levels over the WT NLGN3 control (Figure 4A and Supplementary Figure S2A). Although PERK mediates the phosphorylation of eIF2α upon ER stress activation [25], other types of cellular stress can lead to eIF2α phosphorylation by different kinases in mammalian cells [26]. To study whether the peak of p-eIF2α was due to the activation of PERK, we made use of the highly selective PERK inhibitor GSK2606414 [27]. We first performed a dose–response experiment in parental PC12 cells treated with tunicamycin and thapsigargin to induce canonical UPR and using GSK2606414 at different concentrations, and chose 50 nM as the best condition to observe attenuation of eIF2α phosphorylation (Supplementary Figure S2B). Densitometry quantification of Western blot analysis showed that the GSK2606414 treatment significantly reduced the signal of p-eIF2α in PC12 cells expressing the R451C and G221R mutant proteins for 12 h after NLGN3 induction, whereas no reduction was observed for cells expressing WT NLGN3 (Figure 4B). This supports a causal link between the retention of the mutant NLGN3 variants in the ER and the activation of the PERK branch of the UPR.

### Expression of mutant R451C and G221R NLGN3 causes up-regulation of UPR target genes

**Figure 5:**
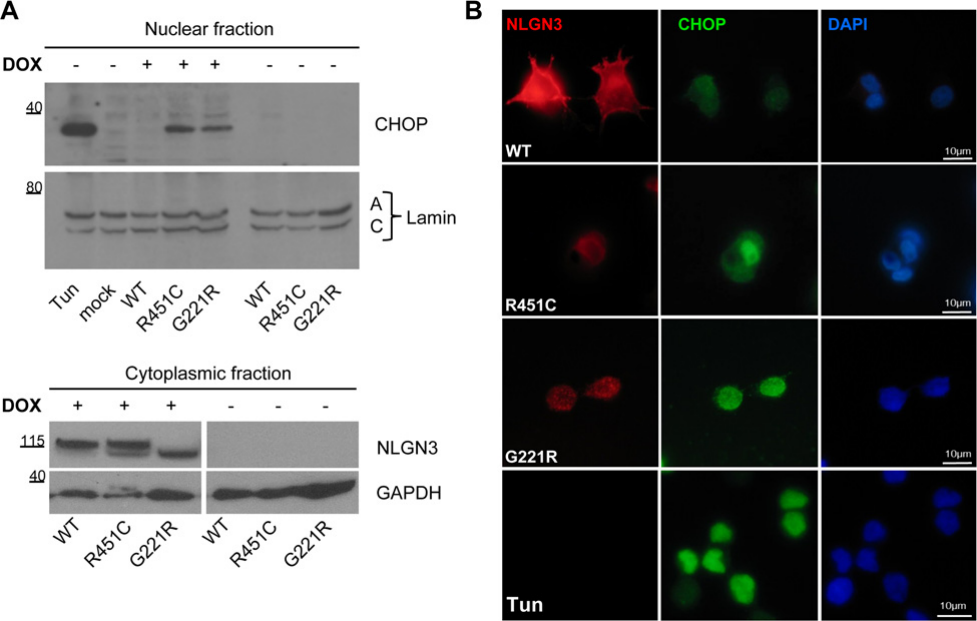
Expression levels of the UPR target CHOP (**A**) Nuclear and cytoplasmic fractions extracted from PC12 NLGN3 cells at 24 h after DOX administration. Upper panel: Western blot detection of CHOP. Parental PC12 cells (mock) and the −DOX conditions were used as negative controls and tunicamycin was used as a positive control. Nuclear fraction was normalized to lamin A/C protein. The Western blot is representative of four independent experiments. Lower panel: NLGN3 detection in the cytoplasmic protein fraction. GAPDH was used as a loading control. Image presents two different parts of the same Western blot. Molecular masses are indicated in kDa. (**B**) Differentiated PC12 NLGN3 cells stained with the anti-CHOP antibody (green) and anti-FLAG for detection of NLGN3 (red). Tunicamycin-treated parental PC12 cells were used as a positive control (lower panel).

After finding that NLGN3 mutant proteins activated all of the UPR stress sensors, we analysed the steady-state levels of UPR target genes such as CHOP and BiP. Under ER stress conditions, the transcription factor CHOP is synthesized and plays a role in regulating ER client protein load and the redox conditions in the organelle [28]. We found that CHOP was induced upon expression of both NLGN3 mutant proteins, as shown by Western blot analysis of nuclear extracts from R451C and G221R NLGN3-expressing cells (Figure 5A, upper and lower panels), but not in cells expressing WT NLGN3 or in cells not treated with DOX (Figure 5A, upper panel). These results were confirmed by immunohistochemical analysis using the same antibody against CHOP (Figure 5B). Whereas the majority of cells overexpressing R451C and G221R NLGN3, and the positive control cells treated with tunicamycin, showed specific nuclear staining for CHOP, the same was not observed in cells expressing WT NLGN3 (Figure 5B).

**Figure 6:**
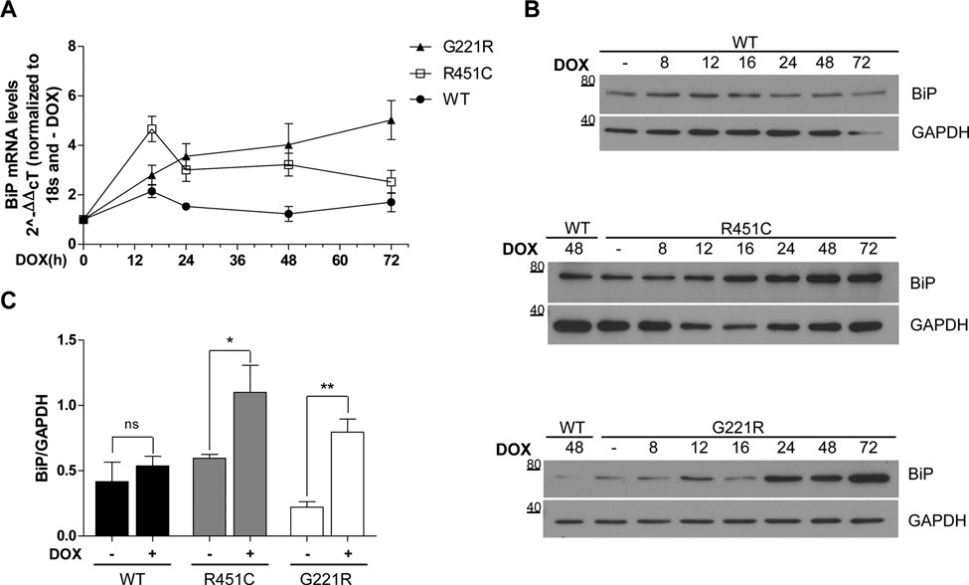
Up-regulation of the UPR target BiP (**A**) NLGN3 was induced from 0 to 72 h by DOX and BiP mRNA levels were quantified. Time point values were normalized to −DOX, for each clone, and statistically compared with WT. Results are means±S.E.M. for four independent experiments. At 16 h from DOX, a robust increase in BiP mRNA is observed in the NLGN3 R451C (**P<0.01) with respect to WT and is still significant at 48 h (*P<0.05). The G221R mutant protein caused an increase over the time becoming gradually significant from 24 to 72 h (*P<0.05 at 24 h, **P<0.01 at 48 h, ***P<0.001 at 72 h). BiP mRNA levels showed a flat trend for the WT NLGN3 clone. Comparison between R451C and G221R revealed significant differences between the two mutants at 72 h, *P<0.05. (**B**) Representative time course of BiP protein levels after DOX treatment from 0 to 72 h. Molecular masses are indicated in kDa. (**C**) Densitometry of BiP normalized to GAPDH levels was calculated after inducing NLGN3 synthesis for 24 h (*n*=4). Statistical analysis compared the +DOX with the −DOX conditions (R451C *P<0.05, G221R **P<0.01).

It is well established that induction of BiP is a marker of ER stress and a central modulator of the UPR [29]. We thus quantified the mRNA levels for BiP in a time course experiment ranging from 0 to 72 h after NLGN3 protein synthesis was induced by DOX. Although WT NLGN3 did not alter the expression of BiP over time (Figure 6A, black circles), the synthesis of R451C NLGN3 caused a robust increase in BiP mRNA at 16 h, followed by a progressive attenuation at 24 and 48 h, decreasing towards WT values at 72 h (Figure 6A, open squares). In contrast, a slower but progressive increase was detected for G221R NLGN3 throughout the entire experimental period (Figure 6A, black triangles).

To investigate further the effects of the NLGN3 mutant proteins on the up-regulation of BiP, we measured its protein levels at several times following DOX treatment. Whereas the expression of BiP was unchanged after WT NLGN3 induction, both mutant NLGN3 variants caused a progressive up-regulation of BiP protein levels when compared with the uninduced condition (Figure 6B). Interestingly, induction of G221R NLGN3 led to an increase in BiP levels that followed the same trend observed for its mRNA (Figure 6A). In the case of the R451C mutant BiP protein levels remained elevated at later time points in contrast with the levels of its mRNA (Figure 6B). Quantification by densitometry of BiP normalized to GAPDH levels at the 24 h time point showed a significant increase in BiP levels in cells expressing mutant, but not WT NLGN3 proteins (Figure 6C).

**Figure 7:**
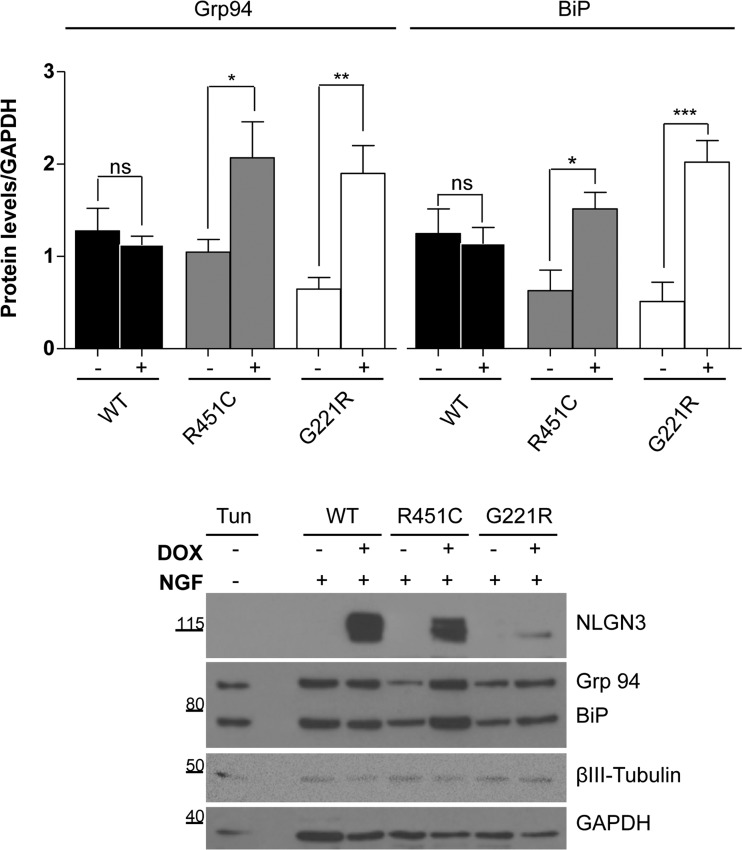
Levels of Grp94 and BiP in differentiated PC12 NLGN3 Tet-On clones Upper panel: densitometry of Grp94 (left) and BiP (right) levels in mutant and WT cells treated with NGF and DOX for 72 h (*n*=3). Statistical analysis compared values of the +DOX condition with the −DOX condition (*P<0.05, **P<0.01, ***P<0.001). Lower panel: representative Western blot of NGF-differentiated PC12 cells for the detection of Grp94 and BiP using the anti-KDEL antibody. βIII-Tubulin was used as a marker for differentiation and GAPDH was used as a loading control. Undifferentiated parental PC12 cells treated with tunicamycin were used as positive control. Molecular masses are indicated in kDa.

We also quantified the protein levels for Grp94 (glucose-regulated protein of 94 kDa) and BiP in PC12 cells differentiated to a neuronal phenotype by treatment with NGF and induced for NLGN3 expression. The efficacy of the NGF treatment was confirmed by βIII-tubulin detection, whereas levels of both chaperones were analysed with an anti-KDEL antibody (Figure 7, lower panel). As expected, densitometry analysis showed a significant increase in Grp94 and BiP levels for both R451C and G221R NLGN3-expressing cells, but not for the control WT NLGN3 cells (Figure 7). These results are in agreement with our previous data showing that BiP and Grp94 were the main chaperones interacting with R451C and G221R NLGN3 [8].

## DISCUSSION

A growing number of neurological genetic diseases are characterized by the accumulation of misfolded proteins within the ER [30]. This can lead to the perturbation of ER homoeostasis and to the activation of the UPR, an adaptive cellular signalling pathway that aims to restore proper functioning of the organelle through the modulation of gene expression [31]. Several mutations in genes coding for synaptic proteins have been identified in ASD patients, suggesting that abnormal synaptic function is a risk factor for neurodevelopmental disorders [32]. The current knowledge regarding the potential link between ASDs and ER stress is minimal and mainly based on autism-associated mutations in *CNTNAP2* [33] and the synaptic adhesion protein *CADM1* [34].

In the present study, we focused on the R451C variant of NLGN3, which is the best characterized autism-linked mutation in the neuroligin family. Our previous work showed that the R451C substitution affects the local folding of the extracellular domain, causing partial retention of the mutant protein within the ER [8]. This misfolding results in a diminished trafficking of the protein to the cell surface, which explains, in part, the reduced effectiveness of R451C NLGN3 to induce clustering of proteins at the synapse in cultured neurons [6]. We have previously compared the R451C mutation with the G221R substitution, which causes global misfolding and complete ER retention of NLGN3 [8,16,35]. Moreover, a substitution homologous with G221R in NLGN3, the G2300R mutation in the C-terminal region of Tg (G2300R), a domain that structurally resembles the extracellular domain of the NLGNs, leads to UPR activation in human and rat tissues [36].

Our newly generated PC12 Tet-On inducible model system allowed us to study the UPR signalling in time course experiments at early and late times after induction of NLGN3 synthesis. Expression of either R451C or G221R mutant NLGN3 caused activation of the transcriptional factors of the UPR over the levels observed after overexpression of the WT protein, including the ER stress sensor ATF6 and the spliced form of the transcription factor XBP1. The kinetics of the UPR response evoked by each mutant variant correlated with the impact that each mutation has on the folding of NLGN3, supporting a direct link between the mutant genotype and the phenotype of the cellular response. In fact, for ATF6 and XBP1 signalling, the different trends reflect the severe and milder effects of the G221R and R451C mutations respectively in the folding of NLGN3 due to their position in the protein's structure [37], with the R451C substitution being more superficial and the G221R one buried in the core of the extracellular domain. The impact of these mutations on the protein's folding is also reflected in the different glycosylation states of the two NLGN3 variants: whereas G221R NLGN3 undergoes a progressive accumulation of high-mannose species in the ER over time, a small fraction of the R451C protein completes its maturation and reaches the cell surface as an EndoH-resistant mature glycoprotein. The existence of two fractions of R451C NLGN3 may explain the observation of a sudden increase in UPR signalling at 16 h that decreased with time (48–72 h).

We also observed activation of the PERK branch of the UPR as increased eIF2α phosphorylation at 12 h after inducing the expression of mutant but not WT NLGN3. Since eIF2α can be phosphorylated via different pathways of the integrated stress response [22], the possibility existed that the phosphorylation we observed was not triggered by PERK. Recently, a number of small molecules have been identified for inhibiting key mediators of ER stress signalling [38,39]. We used GSK2606414 for selectively inhibiting PERK and showed a significant decrease in eIF2α phosphorylation upon R451C or G221R NLGN3 overexpression, supporting a direct involvement of PERK in the phosphorylation of eIF2α.

Overexpression of R451C or G221R, but not WT, NLGN3 in our PC12 cells led to synthesis of CHOP, which is not generally expressed under physiological conditions [40]. Furthermore, we observed a transient increase in BiP mRNA levels upon expression of R451C and G221R NLGN3 that resulted in higher protein levels in both undifferentiated and neuron-like differentiated PC12 cells.

The downstream effects of UPR signalling observed in our PC12 NLGN3 cells when differentiated to a neuronal phenotype suggests that UPR elicited by the ER retention of misfolded R451C NLGN3 might play a role in neuronal behaviour. In fact, although the UPR is classically linked to protein folding stress under pathological conditions, it is becoming clear that UPR signalling also regulates various processes, including synaptic functions [41]. At the molecular level, subtype-selective modulation of cell-surface receptors by CHOP has been reported [42], and the phosphorylation of eIF2α has been associated with synaptic plasticity, learning and memory [43,44]. Moreover, *in vivo* administration of GSK2606414 has been shown to affect memory consolidation, supporting a role for UPR and its mediators in mediating synaptic functions [45].

In conclusion, we show that in our inducible PC12 model system, partial misfolding caused by the R451C autism-linked mutation in NLGN3 activated the UPR. This activation was transient, time-dependent and partially correlated to the severity of the structural alteration caused by this mutation. Our data in PC12 Tet-On NLGN3 cells represent the first detailed evidence on UPR signalling promoted by autism-linked mutations that cause ER retention of the synaptic proteins NLGNs.

It has been reported that a mouse expressing R451C NLGN3 as an endogenous protein exhibits social behavioural traits typical of autism, along with neurotransmission alterations, not observed in the NLGN3-knockout mice [9,46,47]. This gain of function could be explained by the residual fraction of R451C NLGN3 protein that reaching the surface can interact with ligands other than the neurexins. It has been shown that the mutant protein that reaches the surface binds to β-neurexin1 with weaker affinity [4], therefore it is possible that β-neurexin1 is able to associate with other synaptic partners (e.g. LRRTM2), thus driving the gain of function observed in the knockin mice. However, this hypothesis does not exclude the possibility that ER stress, induced by the fraction of mutant NLGN3 that remains within the ER, might be an additional factor taking part in the gain-of-function phenotype.

To gain further insight into the potential role of UPR in neurodevelopmental disorders, it will be critical to test whether the cellular responses observed in our cell model system also take place *in vivo*, and whether the gain of function observed in mice has its origin in these cellular events associated with ER stress.

## Abbreviations

ASD: autism spectrum disorder
ATF6: activating transcription factor 6
BiP: immunoglobulin heavy-chain-binding protein
CHOP: C/EBP (CCAAT/enhancer-binding protein)-homologous protein
DMEM: Dulbecco's modified Eagle's medium
DOX: doxycycline
eIF2α: eukaryotic initiation factor 2α
EndoH: endoglycosidase H
ER: endoplasmic reticulum
ERAD: ER-associated degradation
ERSD: ER storage disease
GAPDH: glyceraldehyde-3-phosphate dehydrogenase
Grp94: glucose-regulated protein of 94 kDa
HRP: horseradish peroxidase
IRE1: inositol-requiring enzyme 1
NGF: nerve growth factor
NLGN: neuroligin
PERK: PKR (dsRNA-dependent protein kinase)-like endoplasmic reticulum kinase
RT: reverse transcription
Tg: thyroglobulin
UPR: unfolded protein response
WT: wild-type
XBP1: X-box-binding protein 1
XBP1s: spliced XBP1
